# Metastatic Thymic Carcinoma with Long Survival After Treatment with Sunitinib

**DOI:** 10.7759/cureus.2982

**Published:** 2018-07-14

**Authors:** Luis Cabezón-Gutiérrez, Parham Khosravi-Shahi, Sara Custodio-Cabello, Maria García-Martos, Magda Palka-Kotlowska, Ana Isabel Franco-Moreno

**Affiliations:** 1 Medical Oncology, Hospital Universitario De Torrejon, Madrid, ESP; 2 Medical Oncology, Hospital Universitario Gregorio Marañon, Madrid, ESP; 3 Medical Oncology, Hospital Universitario De Torrejón, Madrid, ESP; 4 Pathology, Hospital Universitario De Torrejón, Madrid, ESP; 5 Internal Medicine, Hospital Universitario De Torrejón, Madrid, ESP

**Keywords:** thymic carcinoma, sunitinib, metastatic

## Abstract

Thymic carcinomas are the most aggressive histological subtype of thymic tumors with limited data to guide correct management. No standard treatments are available for patients with advanced thymic carcinoma after progressing while on platinum-based chemotherapy. We present a case of a patient with metastatic thymic carcinoma with an unusual response and favorable evolution after receiving treatment with sunitinib, obtaining a progression-free survival of 23 months, much higher than reported to date. We review the literature on the efficacy of sunitinib in metastatic thymic carcinoma after progression to first-line treatment with platinum combinations.

## Introduction

Thymomas account for about 20% of mediastinal neoplasms. Most thymoma patients are between 40 and 60 years of age, and there is a similar incidence in men and women [1]. There are no known risk factors, although, in many cases, thymic carcinomas can be associated with myasthenia gravis and different paraneoplastic syndromes.

Thymomas and thymic carcinomas typically present as an incidental finding, identified on imaging in an asymptomatic patient because of local symptoms (chest pain, cough, shortness of breath, superior vena cava syndrome), or due to symptoms from a paraneoplastic syndrome.

Thymic carcinomas are rare epithelial tumors of the thymus with limited data to guide management decisions and they are the most aggressive thymic tumor subtype as well [2]. It is an aggressive mediastinal neoplasm that arises from the epithelial cells in the thymus gland, with high capacity for local and distant invasion. Therefore, complete resection of these tumors is complicated and in many cases, incomplete. Extrathoracic metastases are seen in fewer than 7% of patients at presentation, most commonly to the liver, kidney, brain, extrathoracic lymph nodes, thyroid, and bone [3].

The management of thymic epithelial tumors is a paradigm of cooperation between clinicians, surgeons, and pathologists, from establishing the diagnosis to organizing the multimodal therapeutic strategy [4].

## Case presentation

A 64-year-old woman with multinodular goiter and depressive disorder with no other associated comorbidities presented to the internal medicine department with facial swelling, dyspnea of moderate exertion, and a feeling of pressure on the face and chest for one month. She presented with no skin lesions or fever.

The physical examination revealed a slight degree of superior vena cava syndrome (SVCS), with facial edema and flushing of the cheeks, and edema of the upper limbs, with a slight increase in jugular venous pressure.

**Figure 1:**
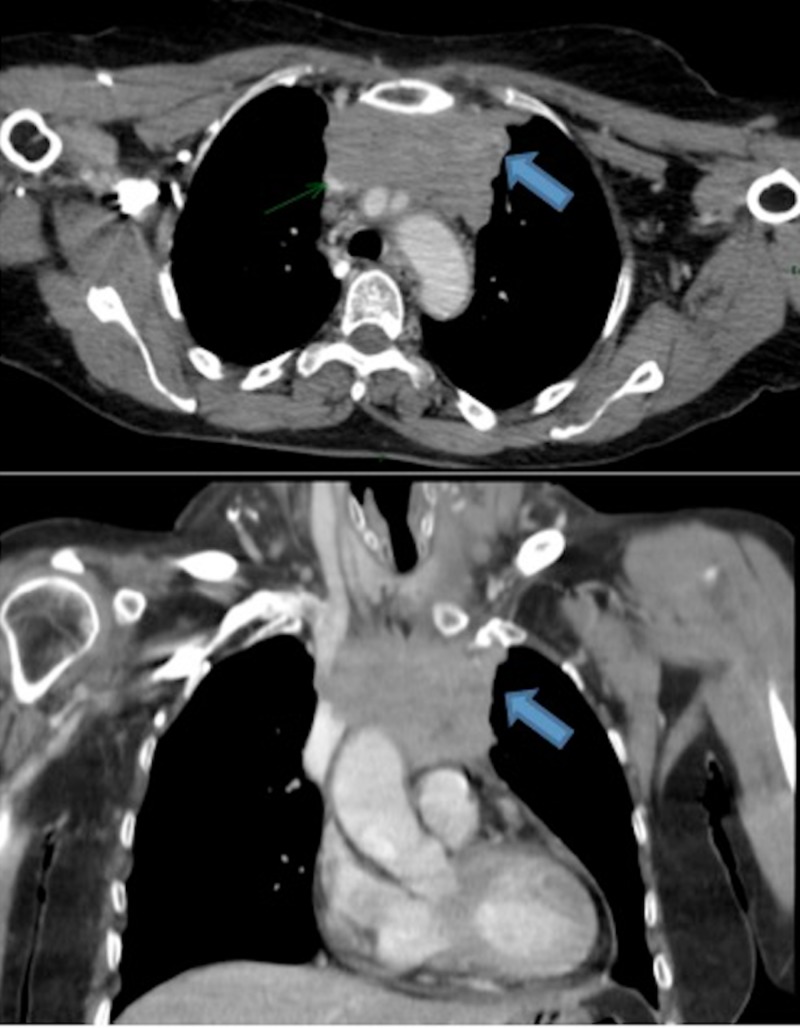
Abdominal-thoracic-cervical CT scan of April 2014. Large, predominantly homogenous mass (discrete left necrotic areas) in the anterior mediastinum with a certain peripheral nodulation that invades the superior vena cava. CT: computed tomography

An abdominal-thoracic-cervical computer tomography (CT) scan was performed (Figure 1) and showed a large mass in the anterior mediastinum.

**Figure 2:**
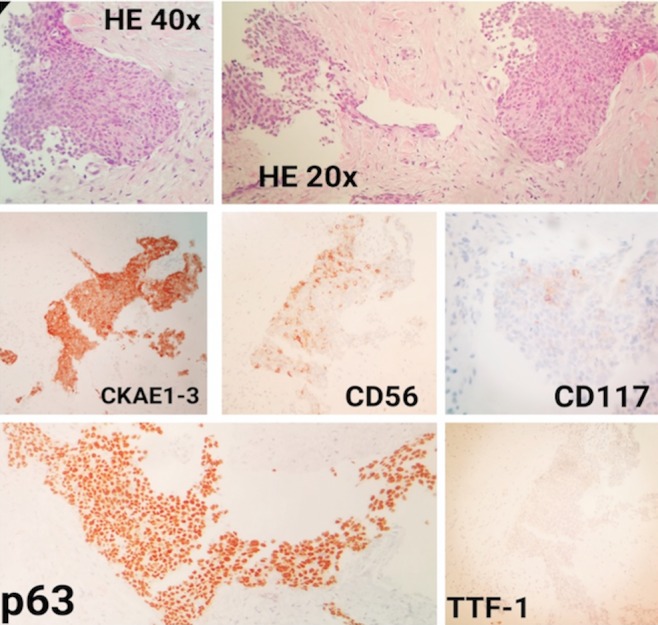
Fibrous and adipose tissue with infiltration by an epithelial neoplasm with areas of tumor necrosis, formed by atypical squamous cells (hematoxylin-eosin (HE) 20x, HE 40x and p63), with a low amount of inflammatory cells and without conclusive evidence of glandular with focal neuroendocrine differentiation (CD56) and poor positivity for CD117 Immunohistochemistry (IHC) study: CKAE1/AE3+, CK7-, CD56+, CK5/6 +, p53+, p63+, calretinin-, TTF-1-, c-kit+ weak, S100-, synaptophysin-; the cell proliferation index of neoplastic cells (ki67) was 20%. Taking these findings into account in correlation with the image, the clinical diagnosis was carcinoma with squamous differentiation.

Computed tomography (CT)-guided biopsy of this mass was performed in April 2014. The diagnosis was compatible with thymic carcinoma (Figure 2). Laboratory tests demonstrated low levels of hemoglobin (11.4 g/dL) and high levels of L-lactate dehydrogenase (622 UI/L). A baseline echocardiogram was performed, repeated every three months, and returned normal.

With a diagnosis of unresectable thymic carcinoma stage III by the Masaoka-Koga system (infiltration of large vessels) and mild superior vena cava syndrome, induction chemotherapy treatment was planned (doxorubicin 40 mg/m2 intravenous (IV) Day 1, cisplatin 40 mg/m2 IV Day 1, vincristine 0.6 mg/m2 IV Day 3, and cyclophosphamide 700 mg/m2 IV Day 4 every three weeks) with corticosteroids (dexamethasone 12 mg daily) for the SVCS.

**Figure 3:**
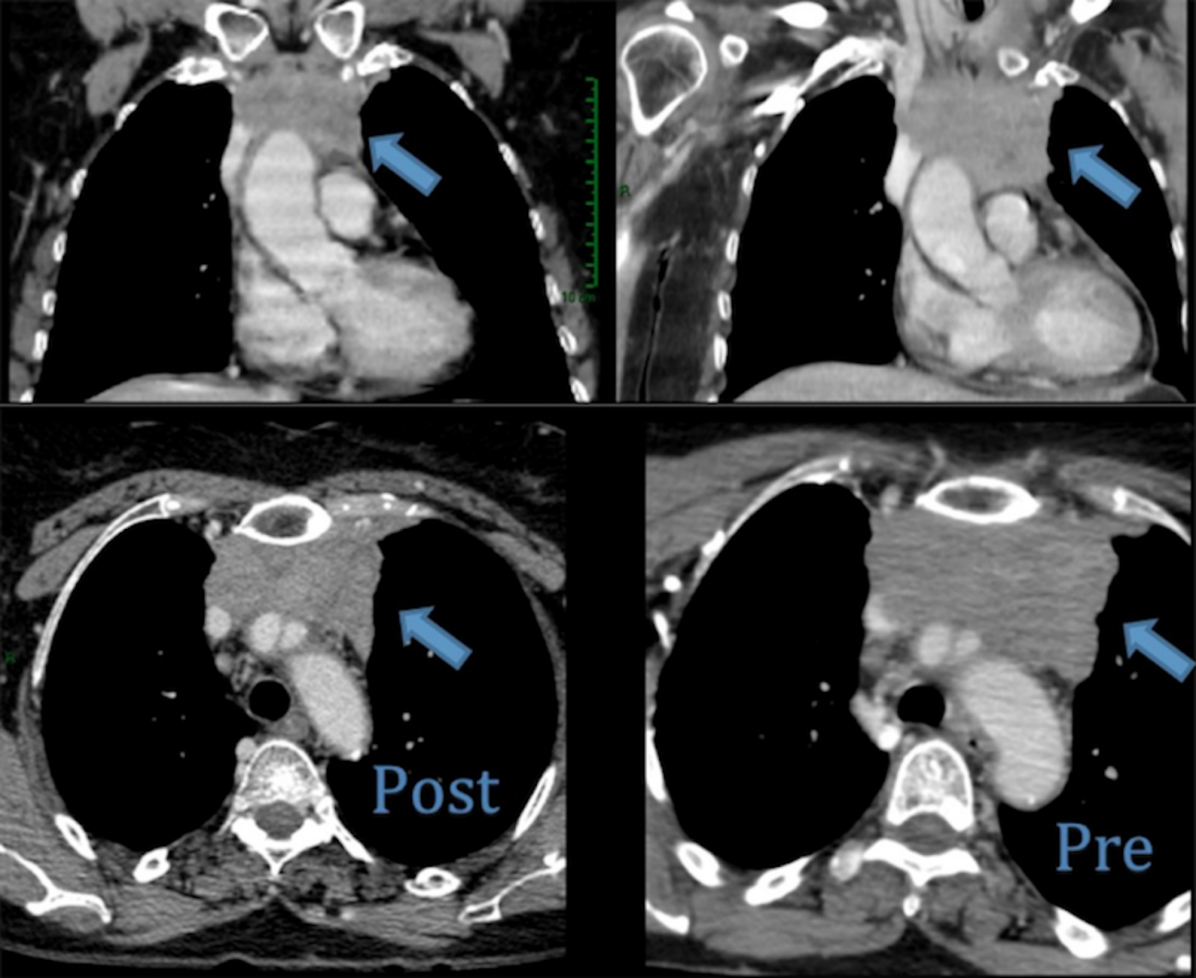
CT scan No significant changes were observed with respect to the previous study, with only a slight decrease in the number of supraclavicular lymph nodes and the size of the mediastinal mass. In the right part of the image (Pre), the thymic carcinoma is shown before the chemotherapy treatment, and in the left part (Post), it is shown after the treatment. CT: computed tomography

After three cycles of chemotherapy, the maximum patient toxicity was grade (G) 2 alopecia, G1 pseudo-influenza syndrome, G1 anemia, and G1 nausea. In the re-evaluation CT performed in July 2014, stabilization of the disease was obtained (Figure 3).

The case was evaluated again in the multidisciplinary oncological committee, and surgical resection was rejected due to the great vessels tumor infiltration. It was decided to administer radical radiotherapy treatment and to undertake a new reassessment of the resectability of the tumor at the end of the treatment.

External beam radiotherapy to the mediastinal mass was administered on tomotherapy, a dose of 5040 cGy in 28 fractions was delivered, and the patient tolerated treatment well. The CT performed two months after the completion of radiotherapy showed stable disease and post-radiotherapy sequelae (G1 pneumonitis). Surgery was definitively dismissed and radiological follow-up was decided. Three months later, pleural and pericardial progression of the disease was observed. A positron emission tomography/CT (PET/CT) scan was performed, confirming the CT findings.

Palliative chemotherapy with a carboplatin area under the curve (AUC) of 5 and paclitaxel 175 mg/m2 IV every 21 days was started. After three cycles, the disease was stabilized, with acceptable tolerability (G1 neurotoxicity, G2 alopecia, G1 nausea, G1 asthenia, G1 arthromyalgia, G2 anemia, G1 thrombocytopenia, and G1 afebrile neutropenia). It was decided to administer a total of six cycles, with a stabilization on CT in September 2015. Follow-up without treatment was started.

**Figure 4:**
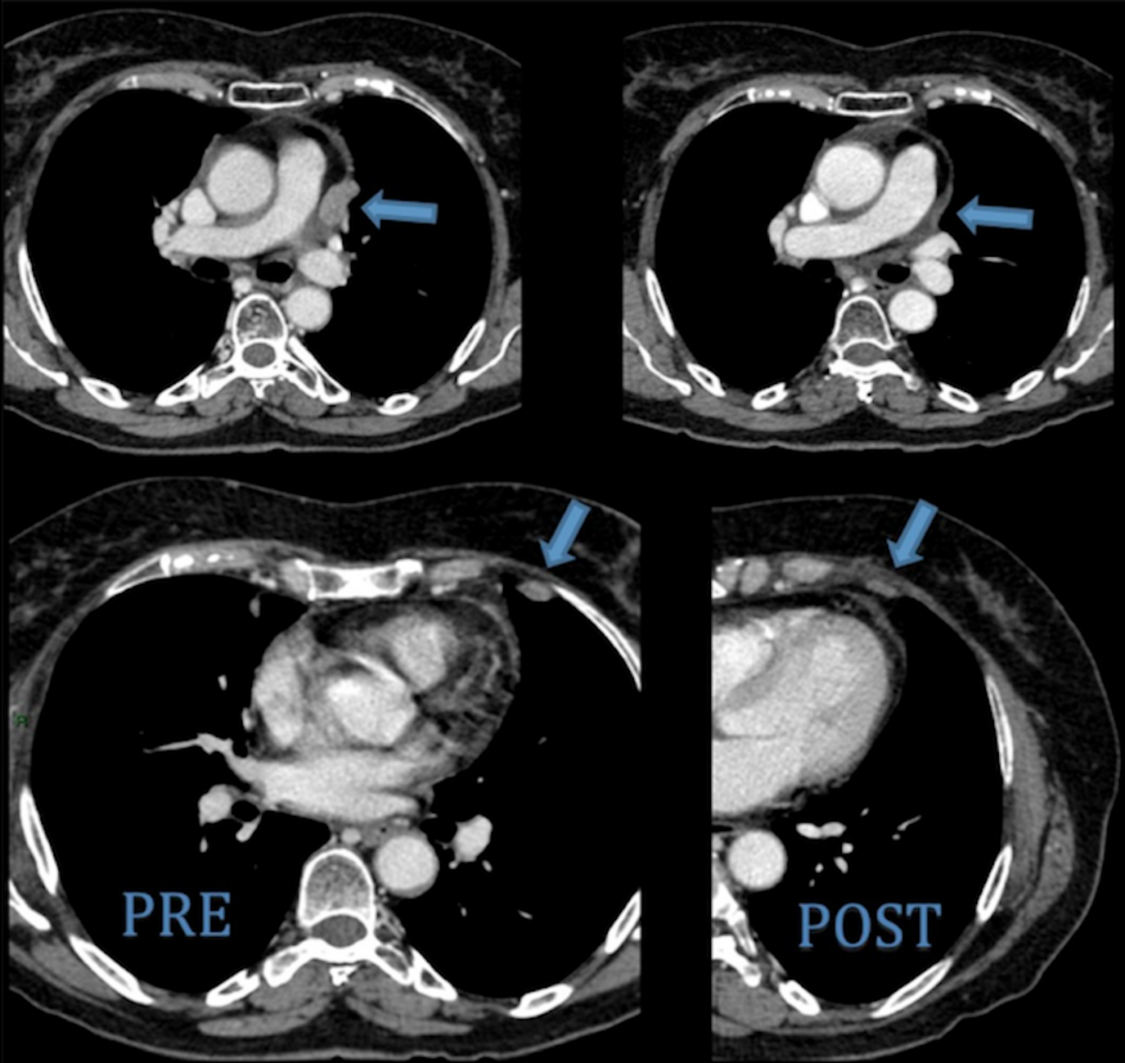
CT scan of May 2016 A significant reduction of pleural and pericardial implants is observed. The comparison between previous (PRE) and posterior (POST) CT to sunitinib treatment is shown. CT: computed tomography

Six months later, the progression of the disease was confirmed by the appearance of new pleural and pericardial implants. At that moment, the patient was asymptomatic, with an Eastern Cooperative Oncology Group (ECOG) scale performance status (PS) of 0 and with a normal echocardiogram and blood test. The second line of palliative treatment was started in January 2016 under compassionate use with 50 mg of sunitinib orally once a day, in six-week cycles (i.e., four weeks of treatment followed by two weeks without treatment). After the first cycle, it was necessary to lower the dose to 37.5 mg per day due to significant toxicity (G2 afebrile neutropenia, G1 thrombocytopenia, G1 hypertension and G3 asthenia), thereafter, her tolerance to sunitinib improved to grade 1 toxicities. Two months after initiating treatment with sunitinib, subclinical hypothyroidism was found, but it was resolved after starting treatment with 50 mcg per day of levothyroxine. After the dose reduction of sunitinib, the patient presented an ECOG PS of 0. After three cycles, a partial response of the disease was obtained (Figure 4).

After 16 cycles of treatment with sunitinib and a progression-free survival (PFS) of 23 months, tumor progression was observed with the appearance of liver metastases in December 2017. The ECOG PS of the patient was 1 at that time. For this reason, in January 2018, the third line of palliative treatment under compassionate use was started with oral capecitabine (650 mg/m2 twice daily on Days 1-14) and gemcitabine (1000 mg/m2 IV on Days 1 and 8) every three weeks. To date, two cycles have been administered with good tolerability (G1 thrombocytopenia, G1 anemia, and G1 asthenia), pending re-evaluation with CT in one month. The current ECOG PS is 1. The overall survival (OS) obtained to date since diagnosis is 48 months.

## Discussion

There are no randomized clinical trials that provide definitive guidance for the management of thymic carcinomas. The International Thymic Malignancies Interest Group (ITMIG) was established to increase collaborative efforts focused on these tumors and develop management guidelines to enhance uniformity in the treatment of these tumors [4].

Surgery is generally considered the main treatment for patients with resectable thymic malignancies and its goal is curative [5]. However, in patients with locally advanced/metastatic thymic carcinomas, the goal is usually to prolong disease-free survival. The decision regarding the role of surgical management in this context should be based on the benefit/risk ratio. The invasion of the lung, pericardium, or certain blood vessels (e.g., innominate vein or the superior vena cava) does not preclude surgical resection. Because surgery is the main treatment modality of thymic carcinoma, all efforts should be made to achieve complete resection.

Chemotherapy is offered in the neoadjuvant setting for those with potentially resectable thymoma or thymic carcinoma or as primary therapy (with or without radiation) for those with unresectable thymoma or thymic carcinoma. Although several regimens are acceptable, we typically use cisplatin, doxorubicin, vincristine, and cyclophosphamide (ADOC)  or cyclophosphamide, doxorubicin, and cisplatin (CAP) for thymic carcinomas in the neoadjuvant chemotherapy setting.

In our case, the patient had an unresectable stage III (Masaoka-Koga system) thymic carcinoma, receiving treatment with induction chemotherapy with the ADOC protocol, although the response to it was not good enough to offer complete microscopic resection (R0) surgery. For this reason, radiotherapy was administered in an attempt to reach enough response to be able to operate. Unfortunately, it was not achieved, and follow-up without treatment was decided. Five months later, the progression of the disease with pericardial and pleural affectation was observed.

The role of chemotherapy and the most active regimen is a discussed topic for treating thymic carcinoma, although the most commonly used therapies contain cisplatin. There are six combinations of chemotherapy regimens for first-line therapy:

- Cisplatin, doxorubicin, and cyclophosphamide (CAP)

- CAP with prednisone

- Cisplatin and etoposide (PE)

- Cisplatin, doxorubicin, vincristine, and cyclophosphamide (ADOC)

- Ifosfamide, cisplatin, and etoposide (VIP)

- Carboplatin and paclitaxel

There are no standard treatments for thymic carcinoma after failure with a first line of platinum-based chemotherapy. There are many single therapeutic agents that have shown some antitumor activity (ifosfamide, etoposide, gemcitabine, pemetrexed, octreotide/prednisone, paclitaxel, and 5-fluorouracil/leucovorin), although their efficacy is limited, with poor results in terms of response rate (RR), PFS, and overall survival (OS).

Many patients, due to their good general condition, are candidates to receive multiple lines of treatment. In this setting, multiple novel agents have been evaluated in thymic malignancies, with variable outcomes [6], including antiangiogenics, targeted agents, and immune-response checkpoint inhibitors (Table 1). In this context, sunitinib is one of the better treatments for pretreated patients.

**Table 1:** Objective response, progression-free survival, and overall survival in thymic carcinomas with selected novel agents N: number of patients; CR: complete response; PR: partial response; SD: stable disease; NR: not reported.

	Study phase	N	Thymic carcinoma	Objective response	Progression-free survival (months)	Overall survival (months)
Sunitinib	II	40	24 (60%)	26% PR 65% SD	7.2	Not reached
Everolimus	II	51	18 (36%)	24% PR 34% SD	5.6	14.7
Cixutumumab	II	49	12 (24%)	0% CR/PR 42% SD	1.7	8.4
Belinostat	II	41	16 (39%)	0% CR/PR 50% SD	2.7	12.4
Lucitanib	Ib	15	12 (80%)	13% PR 73% SD	7.5	NR
Pembrolizumab	II	33	26 (79%)	24% PR 55% SD	6.1	NR

Sunitinib is an oral tyrosine kinase inhibitor, with blocks activity on the vascular endothelial grown factor receptor (VEGFR), proto-oncogene receptor tyrosine kinase (KIT), and platelet-derived growth factor receptor (PDGFR) [13]. Although limited, available evidence suggests that angiogenesis has an important role in thymic epithelial tumors, VEGF is overexpressed in these cancers, and VEGF expression and microvessel density are associated with invasiveness and stage [14]. Higher serum concentrations of VEGF and fibroblast growth factor beta (b-FGF) have been noted in patients with thymic carcinoma [15]. There is an overexpression of KIT in approximately 80% of thymic carcinomas and mutations in the gene encoding this receptor in about 10% of these cancers [16]. PDGF and PDGFRα are also overexpressed in thymic epithelial cells [17].

Thomas et al. in a phase II trial [7] demonstrated the efficacy of sunitinib 50 mg once a day, in six-week cycles (i.e., four weeks of treatment followed by two weeks without treatment) in terms of response and disease control rate in KIT-wild-type thymic carcinomas; overall response rate (ORR) was 26% (with a 91% disease control rate); and median PFS was 7.2 months. OS in thymic carcinomas was not reached. Overall, sunitinib was well-tolerated, with adverse events reported that did not differ substantially from those described in other cancers.

In a retrospective study of 28 patients (20 with thymic carcinoma and eight with thymoma) from the French RYTHMIC network [18], 15 patients (54%) received sunitinib as ≥ fourth-line treatment. The adverse events of sunitinib were all manageable and tolerable. The treatment suspension rate was 28% due to toxicity. Response rate (RR) and median PFS for thymic carcinomas were 22% and 3.3 months, respectively. The median OS in the entire patient population was 15.4 months: survival was not reached for thymoma and was 12.3 months for thymic carcinoma patients (p = 0.043).

In our patient, a partial response and a PFS of 23 months were obtained with sunitinib in the second line, much higher than the 7.2 months obtained in the phase II study by Thomas et al., with mild-moderate toxicity. The OS to date is 25 months, which is much higher than the 12.3 months of median OS obtained in the French retrospective study.

KIT (CD117) is a transmembrane receptor with tyrosine kinase activity and is overexpressed in almost 90% of cases of thymic carcinoma. Its detection by immunohistochemistry is a useful diagnostic tool [16]. c-KIT mutation is observed in 10% of thymic carcinomas [16] and could predict the efficacy of KIT kinase inhibitors (like sunitinib), with some variability based on the type of mutation and the inhibitor [19]. In the patient tumor sample, the immunohistochemical expression of CD117 was weak and the mutation of *c-KIT* was not requested, although the benefit with sunitinib was evident. These results are in accordance with the French retrospective study [18], in which the presence of the c-KIT mutation did not have a clear predictive relationship with sunitinib activity.

In an open-label, nonrandomized, Italian, multicenter [20], phase II study, a total of 15 patients with metastatic thymic epithelial tumors were enrolled in the first stage of a phase II study. Only three patients (20%) were diagnosed with metastatic thymic carcinoma. All patients received oral capecitabine (650 mg/mq twice daily on Days 1–14) and IV gemcitabine (1000 mg/mq on Days 1 and 8) every three weeks. Grades 1–2 neutropenia, anemia, and thrombocytopenia were the most common side-effects, and the most common grade 3 toxicity was neutropenia in three patients (20%). RR in thymic carcinoma was 33% and stable disease in the other 33%. Median PFS was 11 months (six months in the thymic carcinoma subgroup) and median OS time had not been reached.  One- and two-year survival rates were 80% and 67%, respectively (in the overall population).

Based on these results and the non-existence of a standard treatment in the third line of palliative chemotherapy of metastatic thymic carcinoma, the treatment of gemcitabine plus capecitabine was started in January 2018. The efficacy of this treatment has not been reassessed. Tolerability has been good, not exceeding grade 2. At the last visit, the patient maintained an ECOG PS of 1 and carried out a normal life.

## Conclusions

In conclusion, sunitinib may be an option after progression to first-line treatment with platinum combinations therapy for thymic carcinomas. Better knowledge of the molecular mechanisms involved in thymic carcinogenesis could contribute to the development of new and better therapeutic strategies. We have not been able to identify an altered specific oncogenic pathway in the tumor sample that could explain these good results. It will be of great importance in the future regarding the molecular data that next generation sequencing (NGS) can provide and its possible predictive role of response to the different antineoplastic drugs. Prospective randomized trials will be required to provide insight into the most effective option after disease progression with a cisplatin regimen.
